# A retrospective case-control study to evaluate the use of beta-lactam desensitization in the management of penicillin-allergic patients: a potential strategy for Antimicrobial Stewardship Programs

**DOI:** 10.3389/fphar.2023.1260632

**Published:** 2023-11-15

**Authors:** Alicia Rodríguez-Alarcón, Manuela Sanz de Mena, Soukaina Sara Alanti, Daniel Echeverría-Esnal, Luisa Sorli, Elena Sendra, Adela Benítez-Cano, Estela Membrilla, Francesc Cots, Robert Güerri-Fernández, Ramón Adalia, Juan Pablo Horcajada, Fernando Escolano, Santiago Grau, Silvia Gómez-Zorrilla

**Affiliations:** ^1^ Pharmacy Service, Hospital del Mar, Infectious Pathology and Antimicrobial Research Group (IPAR), Hospital del Mar Research Institute, Universitat Autònoma de Barcelona (UAB), Universitat Pompeu Fabra (UPF), Barcelona, Spain; ^2^ Infectious Diseases Service, Hospital del Mar, Infectious Pathology and Antimicrobials Research Group (IPAR), Hospital del Mar Research Institute, Universitat Autònoma de Barcelona (UAB), Universitat Pompeu Fabra (UPF), Barcelona, Spain; ^3^ Center for Biomedical Research in Infectious Diseases Network (CIBERINFEC), Instituto de Salud Carlos III, Madrid, Spain; ^4^ Department of Anesthesiology and Surgical Intensive Care, Hospital del Mar, Parc de Salut Mar. Hospital del Mar Research Institute, Barcelona, Spain; ^5^ Surgery Service, Parc de Salut Mar. IHospital del Mar Research Institute, Barcelona, Spain; ^6^ Management Control Department, Hospital del Mar-Parc de Salut Mar, Barcelona, Spain

**Keywords:** antibiotic desensitization, penicillins, beta-lactams, antimicrobial stewardship programs, hypersensitivity, allergy

## Abstract

**Introduction:** Penicillin allergy labels (PAL) are common in the hospital setting and are associated with worse clinical outcomes. Desensitization can be a useful strategy for allergic patients when alternative options are suboptimal or not available. The aim was to compare clinical outcomes of patients with PAL managed with antibiotic desensitization vs. those who received alternative non-beta-lactam antibiotic treatments.

**Methods:** A retrospective 3:1 case-control study was performed between 2015–2022. Cases were adult PAL patients with infection who required antibiotic desensitization; controls were PAL patients with infection managed with an alternative antibiotic treatment. Cases and controls were adjusted for age, sex, infection source, and critical or non-critical medical services.

**Results:** Fifty-six patients were included: 14 in the desensitization group, 42 in the control group. Compared to the control group, desensitized PAL patients had more comorbidities, with a higher Charlson index (7.4 vs. 5; *p* = 0.00) and more infections caused by multidrug-resistant (MDR) pathogens (57.1% vs. 28.6%; *p* = 0.05). Thirty-day mortality was 14.3% in the desensitized group, 28.6% in the control group (*p* = 0.24). Clinical cure occurred in 71.4% cases and 54.8% controls (*p* = 0.22). Four control patients selected for MDR strains after alternative treatment; selection of MDR strains did not occur in desensitized patients. Five controls had antibiotic-related adverse events, including *Clostridioides difficile* or nephrotoxicity. No antibiotic-related adverse events were found in the study group. In multivariate analysis, no differences between groups were observed for main variables.

**Conclusion:** Desensitization was not associated with worse clinical outcomes, despite more severe patients in this group. Our study suggests that antibiotic desensitization may be a useful Antimicrobial Stewardship tool for the management of selected PAL patients.

## 1 Introduction

Antibiotic hypersensitivity is the most commonly reported class of drug hypersensitivity (Wong et al., 2019; Macy, 2020). A high percentage of the population (15%–20%) carries a penicillin allergy label (PAL) (Blumenthal et al., 2017; Rose et al., 2020). Indeed, penicillin allergy is the leading reported antibiotic allergy and accounts for more than half of all antibiotic allergies (Macy, 2020).

Beta-lactams are among the most commonly prescribed antibiotics with numerous clinical indications and are considered the first-line therapy in many bacterial infections (Categorisation, 2019). Alternative treatments used instead of beta-lactam antibiotics in patients with PAL are less effective, are often associated with a higher frequency of adverse effects, provide unnecessary exposure to broad-spectrum antibiotics with the attendant risk of selection of multidrug-resistant (MDR) microorganisms, and are also associated with increased costs (Blumenthal et al., 2018; Blumenthal et al., 2019a; Castells et al., 2019; Shenoy et al., 2019; Blumenthal et al., 2020).

Published studies exploring the management of PAL patients are increasing (Trubiano et al., 2016; Trubiano et al., 2017a; Trubiano et al., 2017b; Rose et al., 2020; Wijnakker et al., 2023; Stone et al., 2020; Ramsey and Mustafa, 2023). Early de-labelling of PAL patients is strongly supported as one of the main actions in Antimicrobial Stewardship Programs (ASP) to avoid unnecessary use of broad-spectrum antibiotics in low-risk allergic patients and to promote the administration of first-line treatment at an early stage of infection (Wijnakker et al., 2023; Ramsey and Mustafa, 2023). There are some strategies available to remove an allergy label, such as skin tests and oral provocation, as well as obtaining a complete allergy history (Trubiano et al., 2016; Trubiano et al., 2017a; Trubiano et al., 2017b; Rose et al., 2020; Stone et al., 2020). Although less widespread, antibiotic desensitization is an option in patients with confirmed or high-risk penicillin allergy (Habib et al., 2015; Paño-Pardo et al., 2022; Rodríguez-Alarcón et al., 2022). The mechanism of desensitization is to induce tolerance to the drug by administering increasing concentrations of the diluted antibiotic and to prevent anaphylaxis due to inhibition of IgE cross-linking and mast cell degranulation. For this reason, desensitization is indicated only for Ig E-mediated allergic reactions (Blumenthal et al., 2019a; Castells et al., 2019). This strategy is only useful at the time of active infection. When antibiotic treatment is stopped, it should be performed again (Rodríguez-Alarcón et al., 2022).

Prior study suggest that desensitization is well tolerated, even in complex patients with high comorbidity scores (Rodríguez-Alarcón et al., 2022). In clinical practice, the most common indications for desensitization are absence of alternative therapeutic options and therapeutic failure (Rodríguez-Alarcón et al., 2022).

We hypothesized that PAL patients who are managed with alternative non-beta-lactam antibiotic treatments have worse outcomes than those who are desensitized and treated with beta-lactams. To the best of our knowledge, this is the first study to assess this issue in a cohort of PAL patients. Previous studies have noted that prolongation of hospitalization, surgical site infection and treatment failure are costly outcomes that are increased in PAL patients (Huang et al., 2018; Stone et al., 2020). However, studies comparing desensitization to alternative treatments in PAL patients are lacking.

We conducted a 3:1 case-control study to compare the clinical outcomes and mortality of PAL patients with infection who were managed with antibiotic desensitization vs. those who were not desensitized and received alternative non-beta-lactam antibiotic treatment.

## 2 Materials and methods

### 2.1 Study design and participants

A retrospective 3:1 case-control study was conducted at a tertiary care university hospital in Barcelona (Spain), between 2015 and 2022. All adult PAL patients who required antibiotic desensitization for treatment of the infections during the study period were considered cases. In these cases, patients were managed with beta-lactam desensitization and subsequently given a beta-lactam antibiotic. They were identified through electronic medical records. The control group consisted of PAL patients with a clinical infection who required antibiotic therapy and were potentially eligible to receive a desensitization but were managed with alternative antibiotic treatment based on physician decision. The controls were selected per case, adjusted for age, sex, infection source and critical versus non-critical medical services. Controls were included consecutively starting from the initial day of the study period until the required sample size was reached.

Patients with loss to 30-day follow up or with missing data in the study outcomes were excluded. STROBE guidelines were used to report the study (Supplementary Table S1).

### 2.2 Desensitization protocol

An Infectious Diseases physician prescribed desensitization in selected patients. The selection was made from patients with severe or life-threatening infections for whom the physician considered that alternative treatment was not available or was suboptimal and could compromise the patient’s life expectancy due to the source of infection (e.g., central nervous system) or microbiology (Shenoy et al., 2019; Paño-Pardo et al., 2022). In addition, these patients had to meet one of the following criteria: a) patients with confirmed allergy and the results of previous skin tests positive for penicillin; b) a history of immediate hypersensitivity reactions (including anaphylactic reaction); c) PAL with unconfirmed hypersensitivity, but in a compromised clinical situation and in need of penicillin or a penicillin-related antibiotic (Shenoy et al., 2019). These indications were stipulated based on the non-availability of assessment by allergist during the hospitalization of patients in our center.

Since desensitization strategies carry the risk of complications, all patients were transferred to an intensive care unit (ICU) and were strictly monitored and followed during the procedure. The physicians who supervised desensitization were properly trained for the procedure and the nursing team followed a checklist provided by the pharmacy department (Chen et al., 2019; Rodríguez-Alarcón et al., 2022). Desensitization bags were prepared in sterile laminar flow cabinets in the pharmacy, following predefined protocols for each antibiotic (Rodríguez-Alarcón et al., 2022). Desensitization was not associated with a delay in the administration of antibiotic therapy, since the patient received desensitization within the first few hours after it was prescribed. If the procedure had to be delayed, the patient received a single dose of the alternative antibiotic agent before desensitization to avoid delays in antibiotic administration.

### 2.3 Data collection and definitions

Demographic, clinical and epidemiological data were collected from hospital medical and nursing records as follows: age and sex; comorbidities and severity of underlying diseases assessed using the age-adjusted Charlson comorbidity index (Charlson et al., 1987; Charlson et al., 1994). Individual matching of three controls, whenever possible, was used with each case. Case and control groups were adjusted for age, sex, infection source (endocarditis, endovascular, intraabdominal, pancreaticobiliary, skin and soft tissue (SST), respiratory and central nervous system) and critical care vs. non-critical care unit patients. The definitions for specific types of infection were based on Centers for Disease Control and Prevention criteria (CDC/NHSN, 2020). Disease severity was calculated using quick SOFA (Seymour et al., 2016) on the day of the desensitization procedure in the study group, and on the day of initiation of alternative treatment in the control group, adjusted for the previously mentioned criteria. The MDR profile was defined according to current international standard definitions (Magiorakos et al., 2012).

Data on allergy history included date of allergy diagnosis (unknown date of allergy diagnosis, less than 1 year, 1–5 years, more than 10 years, childhood). Date of allergy diagnosis was defined as unknown when a patient had an allergy label but did not remember when they were diagnosed. Medical confirmation of allergy, skin prick and/or intradermal testing, clinical manifestations of allergy and antibiotic involved were also collected. All patients with a delayed-type allergic reaction, such as Toxic epidermal necrolysis (TEN), belonged to the control group, as desensitization is not indicated in these patients because it is not IgE-mediated.

Data on the desensitization procedure (indication, antibiotic involved, duration, completion, reactions) were recorded as previously described (Rodríguez-Alarcón et al., 2022). Desensitization costs were calculated including antibiotic cost plus materials for preparation (bags, serums and syringes), human resources in pharmacy (pharmacy technician) and the cost of a 4-h process in an intensive care unit based on critical unit bed-day cost in Spain which is estimated to be 1,250€/day (Rodríguez Villar and Barrientos Yuste, 2014).

### 2.4 Outcomes and follow-up

The primary outcome variable was clinical cure. Secondary outcomes were 30-day all-cause mortality, infection-related mortality, infection-related hospital days, adverse events related to antibiotic therapy, and hospital readmission at 30 days.

Clinical cure was considered when all signs and symptoms of infection were completely resolved on the day of hospital discharge. Our study included patients with different infectious syndromes, in which length of hospitalization, duration of treatment and/or time to clinical cure can vary depending on the source of infection. For this reason, we performed a second analysis, considering clinical cure at end of treatment (EOT), defined as the resolution of all signs and symptoms of infection at the end of antimicrobial therapy.

Infection-related hospital days was defined as days from the onset of the infection until the end of infection, considered to be discontinuation of antibiotic treatment if the antibiotic was stopped during hospitalization, or hospital discharge if the patient did not finish antibiotic treatment before leaving the hospital.

Hospital readmission was assessed within 30 days of hospital discharge. Patients were followed up to 30 days.

### 2.5 Statistical analysis

It was not possible to calculate the sample size due to the small number of desensitization cases, since desensitization was only prescribed in selected patients. Based on recommendations from studies conducted in rare diseases, the case-control ratio of 1:3 was chosen precisely to increase the precision of the statistical analysis (Iwagami and Shinozaki, 2022). Continuous quantitative variables are presented as means and standard deviation (SD), and categorical variables as number of cases and percentages. The Student’s t-test or Mann-Whitney U test were applied to compare continuous variables, and Fisher’s exact test or Pearson’s χ2 test to contrast categorical variables, as appropriate. All *p*-values were 2-tailed, and statistical significance was set at <0.05. Logistic regression models adjusted for potential confounders were fitted to assess the impact of antibiotic desensitization on outcomes (clinical cure, hospital readmission, mortality). Statistical analysis was performed using SPSS v.25.

### 2.6 Ethical approval

The study design was revised and approved by the Clinical Research Ethical Committee of Parc de Salut Mar (CEIC Parc de Salut Mar, registration no. 2021/9829/I). The need for written consent to participate in the study was waived due to the observational and retrospective nature of the study. However, patients who were desensitized provided written informed consent prior to the desensitization procedure, as required by standard clinical practice.

## 3 Results

Fifty-six patients were included: 14 in the desensitization group and 42 in the control group. The demographic, clinical and epidemiological data are shown in Table 1. The results of desensitization were published in a previous study (Rodríguez-Alarcón et al., 2022).

**TABLE 1:** Demographic, clinical and epidemiological data.

	Desensitized group N = 14	Control group N = 42	*p*-value
Demographic			
Female	9 (64.3)	22 (52.4)	0.32
Male	5 (35.7)	20 (47.6)	0.32
Age, m (SD)	72.8 (±7.5)	73.5 (±13.8)	0.49
**Comorbidities**			
CHARLSON, m (SD)	7.4 (±3.3)	5 (±2.3)	0.01
Diabetes mellitus	5 (35.7)	11 (26.2)	0.36
Respiratory disease	8 (57.1)	11 (26.2)	0.04
Heart disease	6 (42.9)	12 (28.6)	0.25
Chronic kidney disease	5 (35.7)	5 (11.9)	0.05
Liver disease	3 (21.4)	3 (7.1)	0.16
Solid malignancy	5 (35.7)	10 (23.8)	0.29
Haematological neoplasm	0 (0)	1 (2.4)	0.75
Neurological disease	5 (35.7)	4 (9.5)	0.03
**Critical care unit**	5 (35.7)	13 (31)	0.49
**Infection source**			0.78
-Pulmonary	2 (14.3)	11 (26.2)	
-Intraabdominal	3 (21.4)	8 (19)	
-SST	2 (14.3)	7 (16.7)	
-Pancreaticobiliary	1 (7.1)	6 (14.3)	
-Endovascular	2 (14.3)	4 (9.5)	
-Endocarditis	3 (21.4)	3 (7.1)	
-Central nervous system	1 (7.1)	3 (7.1)	
**Infection data**			
Hospital-acquired	5 (35.7)	20 (47.6)	0.32
Community-acquired	9 (64.3)	22 (52.4)	0.32
Post-surgical infection	5 (35.7)	16 (38.1)	0.57
Bloodstream infection	10 (71.4)	22 (52.4)	0.17
QuickSOFA, m (SD)	0.9 (±0.9)	0.9 (±0.9)	0.84
Polymicrobial	6 (42.9)	11 (26.2)	0.19
MDR pathogens^a^	8 (57.1)	12 (28.6)	0.05

Data are presented as *n* (%), unless otherwise specified. SST: skin and soft tissue. quickSOFA: quick Sequential Organ Failure Assessment. MDR: multidrug-resistant.

^a^ At least one MDR pathogen present in cultures. Statistical significance at *p* < 0.05.

No statistically significant differences in allergy data were found between groups: the dates of allergy diagnosis were as follows: unknown 41 patients (73.2%), in childhood in 3 (5.4%), more than 10 years earlier in 5 (8.9%), between 1 and 5 years earlier in 3 (5.4%), and less than 1 year earlier in 4 (7.1%). Previous clinical manifestations associated with the allergy were: unknown 34 (60.7%), rash 13 (23.2%), anaphylaxis 3 (5.4%), others 6 (10.7%) (uvular edema, dizziness, TEN).

The indications for desensitization in the study group were a lack of available alternative treatment options, failure of non-beta-lactam treatment, or the need to optimize treatment in severe infections (Rodríguez-Alarcón et al., 2022).

**FIGURE 1:**
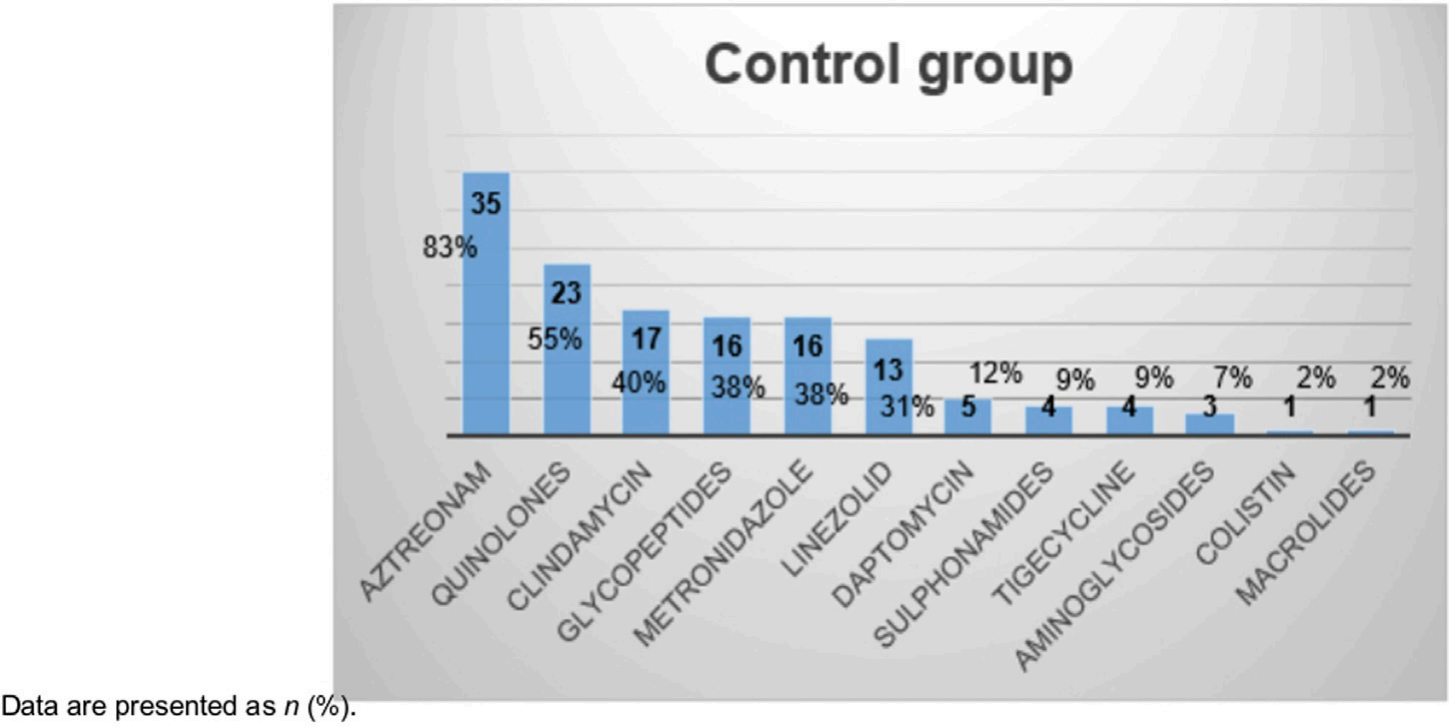
Alternative non-beta-lactam treatment administered to non-desensitized group.

Regarding antibiotic treatment, Figure 1 includes alternative non-beta-lactam treatment received by control group. Table 2 shows differences in primary and secondary outcomes between the two groups.

**TABLE 2:** Primary and secondary outcomes.

	Desensitized group N = 14	Control group N = 42	*p*-value
30-day all-cause mortality	2 (14.3)	12 (28.6)	0.24
In-hospital mortality	2 (14.3)	11 (26.2)	0.30
Infection-related mortality	2 (14.3)	9 (21.4)	0.44
Clinical cure (hospital discharge)	10 (71.4)	23 (54.8)	0.22
30-day hospital readmission	5 (35.7)	7 (16.7)	0.13
Antibiotic-related adverse events	0 (0)	5 (11.9)	0.22

Data are presented as *n* (%), unless otherwise specified. Statistical significance at *p* < 0.05.

Twelve patients were readmitted to hospital within 30 days, and five were infection-related (two in the desensitized group and three in the control group). With respect to selection of MDR strains, none of the desensitized patients who did not previously have a MDR isolate, selected MDR pathogens after being readmitted. In contrast, four patients in the control group who did not previously have MDR pathogens, selected an MDR strain after readmission. Moreover, antibiotic-related adverse events occurred only in the control group. These were: nephrotoxicity in two patients (both related to vancomycin treatment), *Clostridioides difficile* infection in one patient, hepatotoxicity in one patient (related to teicoplanin treatment), and gastrointestinal disorders in one patient (related to levofloxacin treatment).

Univariate and multivariate analysis of the main outcome are shown in Table 3. No differences in clinical cure were observed at hospital discharge between desensitized patients and those who received an alternative treatment after adjusting for potential confounding variables. No statistically significant differences in clinical cure at EOT were found.

**TABLE 3:** Univariate and multivariate analysis of variables predicting clinical cure.

Overall cohort (*n* = 56, clinical cure = 33)						
	Clinical cure (N = 33)	Non-clinical cure (N = 23)	Unadjusted OR (95% CI)	*p*-value	Adjusted OR (95% CI)	*p*-value
Age	73.85 (±12.01)	72.51 (±13.38)	0.97 (0.92–1.03)	0.36	0.98 (0.93–1.03)	0.49
Male	13 (39.4)	12 (52.2)	0.72 (0.19–2.73)	0.63		
CHARLSON index	6.06 (±2.77)	4.96 (±2.69)	1.36 (0.97–1.92)	0.07	1.30 (0.97–1.75)	0.08
Pulmonary disease	11 (33.3)	8 (34.8)	0.57 (0.12–2.79)	0.49		
Chronic kidney disease	5 (15.2)	5 (21.7)	0.17 (0.02–1.28)	0.09	0.24 (0.042–1.43)	0.12
Desensitization	10 (30.3)	4 (17.4)	0.38 (0.07–2.21)	0.28	0.54 (0.11–2.62)	0.45
Post-surgical infection	13 (39.4)	8 (34.8)	1.58 (0.36–6.88)	0.54		
Bloodstream infection	19 (57.6)	13 (56.5)	1.30 (0.30–5.60)	0.72		
QuickSOFA	0.70 (±0.81)	1.22 (±1.08)	0.42 (0.19–0.89)	0.02	0.47 (0.24–0.92)	0.03
Polymicrobial	10 (30.3)	7 (30.4)	0.77 (0.14–4.19)	0.76		
MDR microorganisms	11 (33.3)	9 (39.1)	0.45 (0.09–2.23)	0.33		

Data are presented as *n* (%), unless otherwise specified. OR: odds ratio, CI: confidence interval. quickSOFA: quick Sequential Organ Failure Assessment. MDR: multidrug-resistant. Statistical significance at *p* < 0.05.

For secondary outcomes, 30-day all-cause mortality and 30-day hospital readmission between desensitized patients and the control group were included in the adjusted analysis (Tables 4, 5, respectively).

**TABLE 4:** Univariate and multivariate analysis of variables predicting 30-day all-cause mortality.

Overall cohort (*n* = 56, 30-day all-cause mortality = 14)						
	Mortality (N = 14)	Non-mortality (N = 42)	Unadjusted OR (95% CI)	*p*-value	Adjusted OR (95% CI)	*p*-value
Age, m (SD)	68.05 (±12.8)	75.05 (±12.0)	0.95 (0.89–1.01)	0.10	0.95 (0.89–1.01)	0.09
Male	5 (35.7)	20 (47.6)	0.29 (0.06–1.54)	0.15	0.36 (0.08–1.59)	0.18
CHARLSON index, m (SD)	5.29 (±2.8)	5.71 (±2.8)	1.03 (0.77–1.37)	0.85	1.07 (0.82–1.39)	0.61
Pulmonary disease	4 (28.6)	15 (35.7)	1.34 (0.22–7.99)	0.75		
Chronic kidney disease	3 (21.4)	7 (16.7)	1.94 (0.26–14.35)	0.52		
Desensitization	2 (14.3)	12 (28.6)	3.09 (0.38–25.06)	0.29	2.83 (0.42–19.25)	0.29
Post-surgical infection	6 (42.9)	15 (35.7)	1.33 (0.29–6.06)	0.71		
Bloodstream infection	7 (50.0)	25 (59.5)	0.63 (0.11–3.56)	0.60		
QuickSOFA, m (SD)	1.36 (±1.0)	0.76 (±0.9)	2.04 (0.93–4.46)	0.07	1.82 (0.89–3.68)	0.09
Polymicrobial	3 (21.4)	14 (33.3)	0.67 (0.07–6.22)	0.72		
MDR pathogens	4 (28.6)	16 (38.1)	0.86 (0.10–7.33)	0.89	0.61 (0.13–2.89)	0.53

Data are presented as *n* (%), unless otherwise specified. OR: odds ratio, CI: confidence interval. quickSOFA: quick Sequential Organ Failure Assessment. MDR: multidrug-resistant. Statistical significance at *p* < 0.05.

**TABLE 5:** Univariate and multivariate analysis of variables predicting 30-day hospital readmission.

Overall cohort (*n* = 56, 30-day hospital readmission = 12)						
	Readmission (N = 12)	No readmission (N = 44)	Unadjusted OR (95% CI)	*p*-value	Adjusted OR (95% CI)	*p*-value
Age	77.66 (±7.8)	72.11 (±13.3)	1.04 (0.93–1.17)	0.47		
Male	5 (35.7)	20 (47.6)	0.43 (0.05–3.76)	0.45		
CHARLSON index	8.50 (±3.1)	4.80 (±2.1)	2.29 (1.29–4.07)	0.00	1.91 (1.27–2.86)	0.00
Pulmonary disease	4 (28.6)	15 (35.7)	6.41 (0.63–65.00)	0.12	2.73 (0.50–14.79)	0.24
Chronic kidney disease	3 (21.4)	7 (16.7)	0.19 (0.01–4.28)	0.29	0.45 (0.05–3.94)	0.47
Desensitization	2 (14.3)	12 (28.6)	1.51 (0.06–40.23)	0.80	1.71 (0.20–14.27)	0.62
Post-surgical infection	6 (42.9)	15 (35.7)	0.50 (0.04–5.52)	0.57		
Bloodstream infection	7 (50.0)	25 (59.5)	1.59 (0.13–19.87)	0.72		
QuickSOFA	1.00 (±0.8)	0.89 (±0.9)	0.58 (0.17–1.94)	0.37		
Polymicrobial	3 (21.4)	14 (33.3)	0.24 (0.01–5.64)	0.38		
MDR microorganisms	4 (28.6)	16 (38.1)	0.58 (0.05–6.17)	0.65		

Data are presented as *n* (%), unless otherwise specified. OR: odds ratio, CI: confidence interval. quickSOFA: quick Sequential Organ Failure Assessment. MDR: multidrug-resistant. Statistical significance at *p* < 0.05.

Number of infection-related hospital days were 36.4 (±22.4) in the desensitization group and 16.1 (±17.9) in the control group (*p* = 0.00). In the multiple regression model, there were associations with age (*p* = 0.01) and CHARLSON (*p* < 0.00), but not with desensitization (*p* = 0.36).

The cost of the desensitization procedure was estimated to be approximately 250.50€ per patient. The cost of hospitalization between groups (desensitization and control groups) was not compared.

## 4 Discussion

The present study describes in some detail the clinical characteristics and outcomes of PAL patients with infections who were managed with beta-lactam desensitization followed by beta-lactams compared to those with infection who were treated with alternative antibiotic therapy. In our study, desensitized patients had higher severity of underlying diseases as assessed by the Charlson comorbidity index. It may be assumed that physicians preferred to use a beta-lactam antibiotic in more complicated patients. Nevertheless, the desensitized group was not associated with worse clinical outcomes (clinical cure, mortality, readmissions) in adjusted analysis. Furthermore, desensitized patients had fewer adverse antibiotic events, including *C. difficile* infection and MDR selection.

Desensitization allows patients to receive more optimal treatments with higher clinical cure rates and lower mortality. In a study carried out in 2020, allergy-labelled patients who received alternative treatments were more likely to die in hospital (Krah et al., 2021). Another study evaluating mortality in recorded penicillin allergy found that that label was associated with a 14% increased risk of death, a result that is potentially modifiable by allergy testing to remove the label and better antibiotic prescribing (Blumenthal et al., 2019b). We observed a non-statistically significant trend toward clinical cure in desensitized patients. Mortality rates in our study were almost twice as high in the non-desensitized group (29% vs. 14%), which is consistent with the literature above.

Despite the fact that the desensitized group had more comorbidities, including respiratory, kidney and neurological diseases, the mortality rates in this group were lower and clinical cure was higher, although no statistically significant differences were observed, probably due to the small sample size. The study group had more severe infections, with more bloodstream or polymicrobial infections and MDR microorganisms, the latter with statistically significant differences.

Desensitization was not independently associated with hospital readmission. Duration of hospitalization attributable to infection was longer in the desensitization group; in the adjusted analysis, it was related to age and Charlson index, but desensitization was not independently associated.

It should be noted that, despite the small sample size, the desensitization group did not select for MDR pathogens, whereas the control group did. These data should be interpreted with caution due to the small number amount of patients, but are nevertheless consistent with previous data suggesting that PAL patients treated with alternative antibiotics are more likely to have MDR infections or to be colonized with an MDR organism (Blumenthal et al., 2018; Blumenthal et al., 2019a; Castells et al., 2019; Shenoy et al., 2019; Blumenthal et al., 2020; Stone et al., 2020; Krah et al., 2021). This may be due to the broader spectrum of alternative treatments. In our cohort, we observed that the non-desensitized group frequently received broad-spectrum antibiotics. Our study suggests that this problem could be partially avoided by desensitizing patients with penicillin allergy as part of the activity of ASP.

Another interesting finding is that desensitization did not cause any adverse events related to the desensitized antibiotic, confirming that this strategy is safe. The use of alternative treatments poses a risk of antibiotic-related adverse events such as nephrotoxicity or *C. difficile*, which could complicate the course of infection. Previous studies also suggest that the use of alternative treatments in PAL patients is associated with an increased risk of adverse events (Castells et al., 2019). A high comorbidity index and chronic kidney disease did not induce antibiotic-related adverse events in the study group, which is an important finding because patients with renal insufficiency, who cannot take certain alternative treatments due to their nephrotoxicity, may be a suitable group for desensitization.

The studies carried out so far have been mainly descriptive with small sample sizes (González-García et al., 2021; Rodríguez-Alarcón et al., 2022). Based on current evidence, desensitization should be used as a last resort in the management of PAL patients. The results observed, both in our study and in the literature, provide support that this strategy can be used safely in selected patients. Our study suggests that PAL patients with severe infections for which there are no alternative options available or in whom an alternative antibiotic treatment has failed, may benefit from desensitization. Patients with serious or life-threatening infections in whom the use of alternative antibiotics may be associated with a worse clinical outcome than the use of beta-lactams are also potential candidates for receipt of desensitization. Nevertheless, it should be borne in mind that this is not a risk-free practice since it must be performed in a strictly monitored area such as the ICU, prepared in a sterile cabinet, and the desensitization procedure must be restarted if it is interrupted, or the antibiotic needs to be re-administered. Nevertheless, the time spent in the ICU is usually short (hours) and standardized protocols can be created to prevent certain risks.

In our study, one of the most commonly used antibiotics in the control group was aztreonam, a monobactam class antibiotic with good activity against gram-negative bacteria but has no activity against gram-positive or anaerobic bacteria (Ramsey and MacGowan, 2016). Although aztreonam has a beta-lactam ring, it does not have a bicyclic structure and can be safely administered in patients with penicillin or cephalosporin allergy, with the exception of ceftazidime (Castells et al., 2019). Ceftazidime and aztreonam have identical side chains and clinical cross-reactivity may occur between these antibiotics (Castells et al., 2019; Wijnakker et al., 2023). As aztreonam contains a beta-lactam ring, its inclusion in the control group may have introduced a bias toward better treatment efficacy than if only non-beta-lactam alternative antibiotics had been included. The decision to include aztreonam as an alternative treatment was based on the fact that it is not generally considered a first-line antibiotic (Ramsey and MacGowan, 2016).

The study has certain limitations derived from the fact that the results are based on a retrospective, single-center, case series study. Second, the sample size of the study group was small, due to the prescription of desensitization in selected patients. A 1:3 ratio was selected to increase the precision of the statistical analysis, based on the recommendations of studies applied in rare diseases (Iwagami and Shinozaki, 2022). Future studies with larger samples are required to confirm the optimal methodology to analyze the matter in this study. Third, due to the retrospective nature of the study and the fact that allergy testing was not routinely performed at our center, the penicillin allergy label was not confirmed in all patients, and some of them had an allergy diagnosis of more than 10 years. Confirmation that IgE-mediated hypersensitivity was still present at the time of desensitization was not possible either due to laboratory delays. While we are aware that it would be ideal to confirm allergy prior to desensitization, there are also certain differences between countries in terms of accessibility to allergists (Paño-Pardo et al., 2022), and in the context of a life-threatening infection where the patient is unstable, a full allergy anamnesis is difficult to make. If it is not possible to carry out a thorough allergy evaluation at the time, desensitization is justified even when the allergy label cannot be confirmed, especially in patients with serious and active life-threatening infections, to avoid delaying beta-lactam administration if the patient can realistically benefit from it for the treatment of their infection (Shenoy et al., 2019; Paño-Pardo et al., 2022). Although a good attempt was made to adjust fully for few covariates, the final number of events was low, which restricts the accuracy of some estimates. There were differences in the baseline characteristics between the two study groups; more specifically, the desensitization group had higher comorbidity scores and more drug-resistant infections and these patients were also more likely to be selected for the desensitization procedure. Although it would have been interesting to conduct an economic analysis to compare the groups, this was not possible due to the retrospective nature of the study and the fact that it was impossible to obtain certain data, especially for patients during the COVID-19 pandemics. This is why we only calculated the approximate cost of desensitization.

Several strengths of this study can be highlighted. Recent interventions have focused on the process and outcomes of de-labelling through a thorough history taking, skin testing and oral challenge, but we explored a unique aspect of antibiotic allergy management: desensitization. To our knowledge, this is the first study to compare the clinical characteristics and outcomes of PAL patients treated with beta-lactams after desensitization versus those managed with alternative options. Beta-lactams are the first-line treatment in many bacterial infections and alternative non beta-lactam treatments are often less effective and associated with more adverse effects. This study provided evidence that desensitization was effective, safe, and not associated with worse clinical cure. Due to the small sample size, the results should be interpreted with caution, but the lack of robust studies in the literature makes our study an interesting starting point to present desensitization as an available option to consider in selected PAL patients that is both effective and safe. Finally, antibiotic desensitization was conducted in severely ill patients with high comorbidity scores, some of them with life-threatening infections. Despite this, the desensitization strategy in our cohort proved to be safe, with no adverse events associated with either desensitization or beta-lactams.

We believe that this study can serve as the basis for a prospective study with desensitization as the intervention.

To conclude, despite higher comorbidity scores and MDR infections, desensitization of PAL patients was not associated with worse clinical cure, higher hospital readmissions or higher mortality rates when compared to PAL patients treated with alternative antibiotics. Our results suggest that antibiotic desensitization could be a useful tool in Antimicrobial Stewardship Programs for the management of selected patients allergic to antibiotics.

## Data Availability

The original contributions presented in the study are included in the article/Supplementary Material, further inquiries can be directed to the corresponding author.
