# Supplementary material for: A retrospective case-control study to evaluate the use of beta-lactam desensitization in the management of penicillin-allergic patients: a potential strategy for Antimicrobial Stewardship Programs
Source: Front Pharmacol. 2023 Nov 15;14:1260632. doi: 10.3389/fphar.2023.1260632 (PMC10684946; doi:10.3389/fphar.2023.1260632)
Supplement: Supplementary file 1 [file Table1.DOCX]

Supplementary Material

# Supplementary Table S1

# STROBE Statement—checklist of items that should be included in reports of observational studies

|  | **Item No.** | **Recommendation** | **Assessment in article** | |
| --- | --- | --- | --- | --- |
| **Title and abstract** | 1 | (*a*) Indicate the study’s design with a commonly used term in the title or the abstract | Study design specified in title and abstract | |
|  |  | (*b*) Provide in the abstract an informative and balanced summary of what was done and what was found | Balanced summary included in the abstract | |
| **Introduction** |  |  |  |  |
| Background/rationale | 2 | Explain the scientific background and rationale for the investigation being reported | The scientific background and rationale are included in the Introduction | |
| Objectives | 3 | State specific objectives, including any prespecified hypotheses | The scientific background and rationale are included in the Introduction | |
| **Methods** |  |  |  |  |
| Study design | 4 | Present key elements of study design early in the paper | Study design described in the first part of Methods | |
| Setting | 5 | Describe the setting, locations, and relevant dates, including periods of recruitment, exposure, follow-up, and data collection | Described in Methods | |
| Participants | 6 | 1. *Case-control study*—Give the eligibility criteria, and the sources and methods of case ascertainment and control selection. Give the rationale for the choice of cases and controls 2. Case-control study—For matched studies, give matching criteria and the number of controls per case | Described in Methods  Described in Methods | |
| Variables | 7 | Clearly define all outcomes, exposures, predictors, potential confounders, and effect modifiers.  Give diagnostic criteria, if applicable | Defined in Methods | |
| Data sources/ measurement | 8* | For each variable of interest, give sources of data and details of methods of assessment (measurement). Describe comparability of assessment methods if there is more than one group | Specified in Methods. The same methods for data collection were used in both groups. | |
| Bias | 9 | Describe any efforts to address potential sources of bias | Selection bias: inclusion of consecutive cases. Information bias: use of well defined, standard, easy to collect variables.  Use of hard outcome variables. | |
| Study size | 10 | Explain how the study size was arrived at | This a 3:1 case-control study, thus, sample size was arrived based on the number of available cases | |
| Continued on next page |  |  |  |  |

| Quantitative variables | 11 | Explain how quantitative variables were handled in the analyses. If applicable, describe which groupings were chosen and why | Explained in Methods |
| --- | --- | --- | --- |
| Statistical methods | 12 | (a) Describe all statistical methods, including those used to control for confounding | Included in Methods |
|  |  | (b) Describe any methods used to examine subgroups and interactions Included in Methods | |
|  |  | (c) Explain how missing data were addressed | Patients with missing data in the study outcomes were excluded due to loss to follow up. Variables with >25% missing were not accounted for multivariate analysis. |
|  |  | 1. Case-control study—If applicable, explain how matching of cases and controls was addressed | Included in Methods |
|  |  | (e) Describe any sensitivity analyses Not applicable | |
| Results |  |  | |
| Participants | 13* | (a) Report numbers of individuals at each stage of study—eg numbers potentially eligible, examined for eligibility, confirmed eligible, included in the study, completing follow-up, and analysed | Not included |
|  |  | (b) Give reasons for non-participation at each stage Not included | |
|  |  | (c) Consider use of a flow diagram Not included | |
| Descriptive data | 14* | (a) Give characteristics of study participants (eg demographic, clinical, social) and information on exposures and potential confounders | Included in Results and Table 1. |
|  |  | (b) Indicate number of participants with missing data for each variable of interest No patients with missing data | |
| Outcome data | 15* |  | |
|  |  | Case-control study—Report numbers in each exposure category, or summary measures of exposure Included in Results | |
|  |  |  | |
| Main results | 16 | (a) Give unadjusted estimates and, if applicable, confounder-adjusted estimates and their precision (eg, 95% confidence interval). Make clear which confounders were adjusted for and why they were included | Specified in Results |
|  |  | (b) Report category boundaries when continuous variables were categorized No categorization was made | |
|  |  | (c) If relevant, consider translating estimates of relative risk into absolute risk for a meaningful time  period Not applicable | |

Continued on next page

| Other analyses | 17 | Report other analyses done—eg analyses of subgroups and interactions, and sensitivity analyses Specified in Methods and Results | |
| --- | --- | --- | --- |
| Discussion |  |  | |
| Key results | 18 | Summarise key results with reference to study objectives Included in Abstract and Discussion | |
| Limitations | 19 | Discuss limitations of the study, taking into account sources of potential bias or imprecision. Discuss both direction and magnitude of any potential bias | Included in Discussion |
| Interpretation | 20 | Give a cautious overall interpretation of results considering objectives, limitations, multiplicity of Included in Discussion  analyses, results from similar studies, and other relevant evidence | |
| Generalisability | 21 | Discuss the generalisability (external validity) of the study results Included in Discussion | |
| Other information | | | |
| Funding | 22 | Give the source of funding and the role of the funders for the present study and, if applicable, for the original study on which the present article is based | Included |

*Give information separately for cases and controls in case-control studies and, if applicable, for exposed and unexposed groups in cohort and cross-sectional studies.

Note: An Explanation and Elaboration article discusses each checklist item and gives methodological background and published examples of transparent reporting. The STROBE checklist is best used in conjunction with this article (freely available on the Web sites of PLoS Medicine at [http://www.plosmedicine.org/,](http://www.plosmedicine.org/) Annals of Internal Medicine at [http://www.annals.org/,](http://www.annals.org/) and Epidemiology at [http://www.epidem.com/).](http://www.epidem.com/)) Information on the STROBE Initiative is available at [www.strobe-statement.org.](http://www.strobe-statement.org/)
