# Supplementary material for: A retrospective case-control study to evaluate the use of beta-lactam desensitization in the management of penicillin-allergic patients: a potential strategy for Antimicrobial Stewardship Programs
Source: Front Pharmacol. 2023 Nov 15;14:1260632. doi: 10.3389/fphar.2023.1260632 (PMC10684946; doi:10.3389/fphar.2023.1260632)
Supplement: Supplementary file 2 [file Table2.DOCX]

**Supplementary Material Table S2. Dates of allergy diagnosis between groups.**

|  | **Desensitized group**  **N=14** | **Control group**  **N=42** | **p-value** |
| --- | --- | --- | --- |
| **Allergy diagnosis** |  |  | 0.07 |
| Childhood | 0 (0) | 3 (7.1) |  |
| More than 10 years | 3 (21.4) | 1 (2.4) |  |
| 1-5 years | 1 (7.1) | 1 (2.4) |  |
| Less than 1 year | 2 (14.3) | 2 (4.8) |  |
| Unknown | 8 (57.1) | 35 (83.3) |  |
|  |  |  |  |

Data are presented as *n* (%), unless otherwise specified. Statistical significance at *p* < 0.05
